# Investigating antibiotic resistance in enterococci in Gabonese livestock

**DOI:** 10.14202/vetworld.2022.714-721

**Published:** 2022-03-25

**Authors:** Otsaghe Ekore Desire, Boundenga Larson, Onanga Richard, Mabika Mabika Rolande, Kumulungui Brice Serge

**Affiliations:** 1 Centre International de Recherche Médicales de Franceville, BP: 769, Franceville, Gabon; 2 Ecole Doctorale Régional d’Afrique Central, BP: 876, Franceville, Gabon; 3 Department of Anthropology, Durham University, South Road, Durham, DH1 3LE, UK

**Keywords:** antimicrobial, cattle, enterococci, Gabon, laying hens, sheep, swine

## Abstract

**Background and Aim:**

The emergence of antibiotic resistance is a major problem worldwide. Antibiotics are often used to prevent or treat infections in livestock. This study aimed to investigate antibiotic resistance in enterococci in Gabonese livestock.

**Materials and Methods:**

We collected 174 animal samples (46 laying hens, 24 swine, 62 cattle, and 42 sheep) from farms in four provinces of Gabon. Bacterial strains belonging to the genus *Enterococcus* were obtained using selective media and polymerase chain reaction targeting the *tuf* gene. Antibiotic susceptibility was determined by the disk diffusion method on Mueller-Hinton agar.

**Results:**

Enterococci were present in 160 of the samples (97%), distributed as follows: laying hens (100%, 41/41), swine (100%, 22/22), small ruminants (88%, 37/42), and cattle (100%, 60/60). Resistance to cephalothin/cephalexin, streptomycin, and rifampicin (RIF) was high, and resistance to vancomycin (VAN), erythromycin, and tetracycline was moderate. A high diversity of resistance was found in Haut-Ogooué and Estuaire provinces. Laying hens and swine showed moderate levels of resistance to ciprofloxacin and penicillin, while sheep and cattle had high levels of resistance to RIF. All species showed a high level of resistance to VAN. We found various patterns of multiple resistances in the isolates, and the multiple resistance indexes ranged from 0.2 to 0.8.

**Conclusion:**

This study shows that livestock in Gabon can be considered potential reservoirs of resistance.

## Introduction

The problem of antibiotic resistance plays an important role in the world due to the emergence and dissemination of resistance genes, mainly in human and animal hosts [1]. Antibiotic resistance in livestock is due to the use of antibiotics as therapeutic, prophylactic agents and growth promoters [2,3]. Antibiotic misuse could lead to the emergence of antibiotic-resistant bacteria in livestock and thus create reservoirs of resistance genes [4,5], potentially transmitted to humans through direct or indirect contact [6,7]. However, data on antibiotic resistance in low- and middle-income countries are scarce, making it challenging to establish antibiotic stewardship systems [8]. Thus, studies characterizing antibiotic resistance in animals are essential in these countries.

Antibiotic resistance is most often investigated in *Enterococcus* spp. because of the plasticity of their genome and the persistence of this genus in the environment, which allow it to acquire antibiotic resistance genes and colonize different ecological niches [9]. Enterococci are ubiquitous in the intestinal tract of farm animals but constitute a small proportion of bacterial ecological diversity [10]. In particular, the species *Enterococcus durans*, *Enterococcus hirae*, *Enterococcus gallinarum*, *Enterococcus casseliflavus*, *Enterococcus faecalis*, and *Enterococcus faecium* are often found in the digestive tract of farm animals [11,12]. *Enterococcus* species have intrinsic resistance to aminoglycosides (a low-level), penicillins (a low-level), vancomycins (VANs) (*E. gallinarum* and *E. casseliflavus*), polymyxins, and streptogramins [13]. The presence of other resistances in *Enterococcus* species could be the result of antibiotic use, thus allowing its use as a bacterial model for the evaluation of the selection pressure created by the consumption of antibiotics in livestock.

In Gabon, studies of the presence of antibiotic resistance in hospitals [14-16] and mammals [17] have shown high rates of several families of antibiotics associated with resistance genes. Another study of antibiotic resistance has revealed a high prevalence to ampicillin and cephalosporins in ready-to-eat chickens [18].

However, antibiotic resistance has not been characterized in Gabonese livestock. Such studies are needed to complement the data already available for hospitals and in the environment. Thus, this study aimed to investigate antibiotic resistance in Enterococci in Gabonese livestock.

## Materials and Methods

### Ethical approval and Informed consent

This study was conducted in Gabon and approved by the Gabonese Ministry of Agriculture, Livestock, Fisheries, and Rural Development (General Direction of livestock, Authorization N°0052/SG/DGE). All samples from farm animals were collected after obtaining verbal consent from the farm manager.

### Study period and location

**Figure-1:**
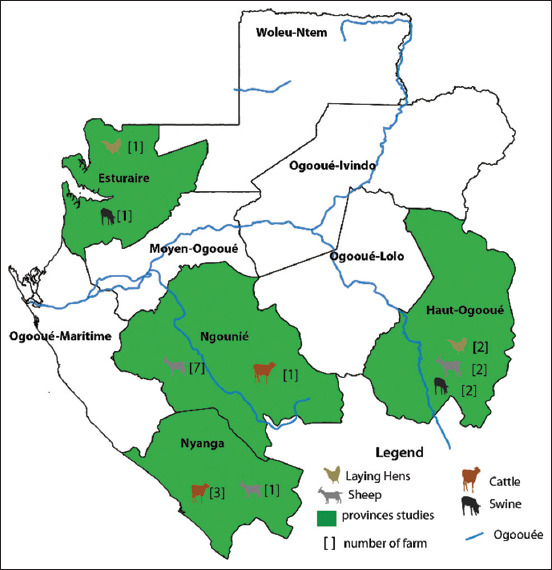
Description of sampling locations. [Source: The authors made the figure with the help of Illustrator CS6 software].

The study was conducted from December 2018 to January 2020. Twenty farms were sampled in four provinces of Gabon (Estuaire, Haut Ogooué, Ngounié, and Nyanga) [Figure-1].

### Fecal sampling

We collected fresh droppings and rectal swabs (3 to 5g) to characterize *Enterococcus* spp. in livestock (chicken, cattle, swine and sheep). The capacity of the farms was as follows: cattle (101-150 (1), 301-350 (1), 1001-1100 (1)), swine (41-60(2), 81-100 (1)), and sheep (11-20 (3), 21-40 (1), 41-60 (1), 61-80(1)). For large populations on farms (e.g., laying hens), 15-20% of the total population was sampled to prevent repeatability during sampling. Each fecal sample was collected on a sterile swab (Copan, France) or in a sterile plastic jar (Qualibacter, France) then hermetically sealed and transported to the Centre International de Recherche Médicale de Franceville bacteriology laboratory for analysis.

### Culture, isolation, and purification of colonies

Each sample was cultured on D-Coccosel (bioMérieux, France) and Slanetz-Bartley (bioMérieux) agars, which are specific culture media for enterococci, at 37°C for 18-24 h. The selection of individual suspect colonies was made according to color and morphology. Black colonies on D-Coccosel (bioMérieux) and white colonies on Slanetz-Bartley (bioMérieux) were grown on an enrichment medium at 37°C for 18-24 h.

### Biochemical identification

Some characteristic colonies obtained were identified using biochemical tests (Gram stain, catalase, and coagulase test), Strep API strips (bioMerieux) to confirm the genus and then stored on phosphate-buffered saline (pH =7.2)/Glycerol (70/30%) at –80°C.

### Molecular identification of selected isolates

DNA was extracted using the Booling method described by Peng *et al*. [19] and quantified using a NanoDrop (Nanovue plus, UK). Genus determination was performed by amplifying a conserved sequence of the *tuf* gene using the primers: 5’- TACTGACAAACCATTCATGATG-3’ and 5’- AACTTCGTCACCAACGCGAAC-3’ described by Iweriebor *et al*. [20]. The polymerase chain reaction (PCR) mix consisted of 3 μL of template DNA and 17 μL of reaction mixture consisting of ×1 buffer, 0.2 mM dNTPs, 2.5 mM MgCl_2_, 0.2 mM of each primer, 50 μL of nuclease-free water, and 0.5 U/mL Taq polymerase for a final volume of 20 μL/tube. The PCR program was 3 min of initial denaturation at 95°C, followed by 35 cycles of denaturation at 95°C for 30 s, hybridization at 55°C for 30 s, elongation at 72°C for 60 s, and final elongation at 72°C for 7 min. The amplicons obtained were revealed after migration by electrophoresis on the 1% agarose gel at 100 V for 40 min with red gel and observed under ultraviolet light (ALLIANCE 4.7 transilluminator Merton, France). After confirming the presence of the required PCR products on the gel, some amplicons were sent to Macrogen (Amsterdam, Pays-Bas) for Sanger sequencing. Analysis and identification of these sequences were carried out online using the BLAST program available on the National Center for Biotechnology Information website (http://www.ncbi.nlm.nih.gov).

### Antibiotic susceptibility testing

Antibiotic susceptibility tests were performed using the Kirby–Bauer disk-diffusion method [21]. Antibiotics tested were chosen according to those used on the farms and those recommended by the Clinical Laboratory Standard Institute [22]. The choice of antibiotics was made according to their use in the farms. Thus, 13 antibiotics were used for laying hens and swine: Erythromycin (ERY, 15 μg), Tetracycline (TET, 30 μg), VAN, 5 μg, Teicoplanin (TEI, 30 μg), Streptomycin (STR, 10 μg), Kanamycin, 30 μg, Cephalothin/cephalexin (CEP, 10 μg), Chloramphenicol (CHL, 30 μg), Ampicillin (AMP, 10 μg), Rifampicin (RIF, 5 μg), Norfloxacin (NOR, 5 μg), and Ciprofloxacin (CIP, 5 μg). Five antibiotics were used for cattle and small ruminants: ERY, 15 μg, TET, 30 μg, RIF, 5 μg, VAN, 5 μg, and TEI, 30 μg.

### Statistical analysis

The multiple antibiotic resistance index (MARI) was calculated following Krumperman [23] and the multidrug resistance (MDR) profile was described as resistance to a minimum of one antibiotic in a minimum of three antimicrobial classes. Statistical analyses were performed using R software (version Ri386 3.5.1, Foundation for Statistical Computing, Vienna, Austria). The Chi-square test was used to test the relationship between province surveyed and prevalence. We considered differences significant at p<0.05.

## Results

### Distribution of Enterococcus spp.

**Figure-2:**
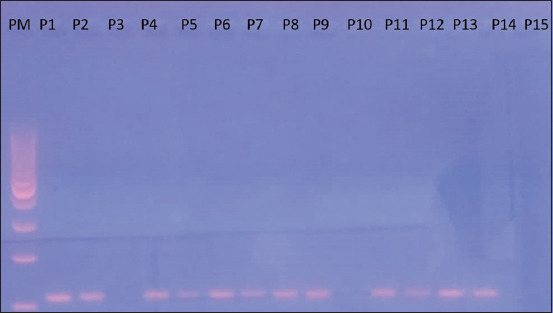
Detection of *tuf* (115pb) gene by polymerase chain reaction, PM: Molecular weight marker, P5: Negative Control, P1: Positive sample, P3: Negative sample.

Enterococci were isolated from 160 (97%) samples, including laying hens (41/41, 100%), swine (22/22, 100%), sheep (37/42, 88%), and cattle (62/62, 100%) (Figure-2). High prevalence occurred in all four provinces: Estuaire (29, 100%), Haut Ogooué (36, 100%), Ngounié (62, 95%), and Nyanga (35, 92%) (Table-1).

**Table-1:** Characteristics of the sample.

Characteristics	Sample	*Enterococcus* spp. (%)	Statistical test
Species			
Laying hens	41	41 (100)	
Swine	22	22 (100)	
Sheep	42	37 (88)	
Cattle	62	62 (100)	
Province			
Estuaire	29	29 (100)	χ^2^=1.8 df=3
Haut-Ogooué	37	36 (100)	p=0.61
Ngounié	65	62 (95)	
Nyanga	35	35 (92)	

### Prevalence of resistance in Enterococci

**Figure-3:**
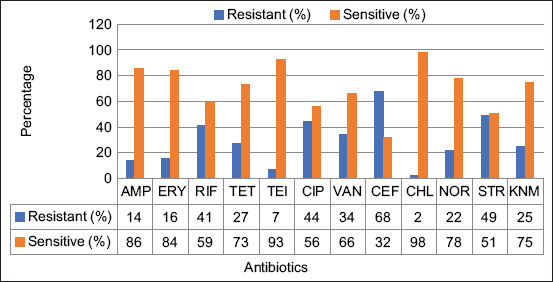
Global distribution of antibiotic resistance.

Isolates showed a high prevalence of resistance to CEP (68%), RIF (57%), CIP (46%), and STR (49%). Moderate prevalence of resistance was found for VAN (21%), NOR (22%), TET (27%), ERY (16%), and AMP (14%). A low prevalence of resistance was found for TEI (7%) and CHL (2%) (Figure-3).

### Resistance per province

**Figure-4:**
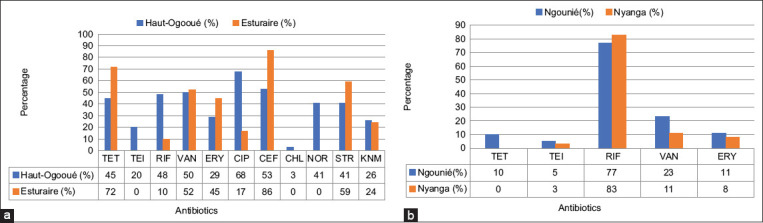
Distribution of resistance by province. (a) Diversity of resistance in Estuaire and Haut Ogoouée, (b) Diversity of resistance in Ngounié and Woleu-Ntem.

Estuaire province had resistance to 9/11 antibiotics tested with the high prevalence of resistance to CEP (86%), TET (72%), STR (59%), VAN (52%), and ERY (45%). In the Haut-Ogoouée, 11/11 of antibiotics tested showed resistance with the high frequencies of resistance for CIP (68%), VAN (50%), TET (45%), and CEP (53%) (Figure-4a). In Nyanga 4/5 and Ngounié 5/5 of the antibiotics tested showed resistance, with a very high frequency of resistance to RIF (83% and 77%, respectively), moderate rates for VAN (11 and 23%), and very low frequency of resistance to ERY (8 and 11%) and TEI (3 and 5%) (Figure-4b).

### Susceptibility to antibiotics by animal species

We found a high prevalence of resistance to TET in laying hens (78%) and swine (41%) but a low prevalence for cattle (8%) and small ruminants (1%). RIF resistance had a high prevalence in small ruminants (67%) and cattle (83%) but a moderate prevalence in laying hens (19%) and swine (36%). Moderate prevalence of resistance to ERY was found for laying hens (39%) and small ruminants (16%), whereas there was a high prevalence of resistance to VAN in swine (95%) and moderate in small ruminants (32%), laying hens (34%), and cattle (20%). Among antibiotics tested in laying hens and swine, CEP resistance was high in swine (100%) and moderate in laying hens (51%). In contrast, a moderate prevalence of resistance was found for penicillin (15 and 14%, respectively, for laying hens and swine). STR and CIP resistance showed moderate prevalence in laying hens (61% and 32%) and swine (27% and 68%) (Table-2).

**Table-2:** Antibiotic resistance of *Enterococcus* isolates from livestock.

Drug	Laying hens n (%)		Swine n (%)		Sheep n (%)		Cattle n (%)	
			
	R	S	R	S	R	S	R	S
Tetracycline	32 (78)	9 (22)	9 (41)	13 (31)	3 (8)	34 (92)	1 (1)	61 (98)
Rifampicin	8 (19)	33 (80)	8 (36)	14 (64)	25 (67)	12 (32)	52 (83)	10 (16)
Erythromycin	16 (39)	25 (60)	0 (0)	22 (100)	6 (16)	31 (84)	4 (7)	57 (93)
Vancomycin	14 (34)	27 (66)	21 (95)	1 (1)	9 (32)	28 (68)	12 (20)	49 (80)
Teicoplanin	7 (17)	34 (83)	1 (1)	21 (95)	1 (1)	36 (97)	3 (1)	59 (95)
Ampicillin	6 (15)	35 (85)	3 (14)	19 (86)	NT	NT	NT	NT
Cephalothin/Cephalexin	21 (51)	20 (49)	22 (100)	0 (0)	NT	NT	NT	NT
Streptomycin	25 (61)	16 (39)	6 (27)	16 (73)	NT	NT	NT	NT
Kanamicin	13 (32)	28 (68)	3 (14)	19 (86)	NT	NT	NT	NT
Chloramphenicol	0 (0)	41 (100)	1 (1)	21 (95)	NT	NT	NT	NT
Ciprofloxacin	13 (32)	28 (68)	15 (68)	7 (32)	NT	NT	NT	NT

NT=Not tested, R=Resistant, S=Sensible

### Multiple antibiotic resistance profile

The MDR profile of the *Enterococcus* revealed resistance to a minimum of three antimicrobial classes with a MARI of 0.2-1 Fifty-two (32%) isolates were resistant to multiple drugs (Tables-3 and 4). Two sheep isolates had a MARI of 0.5-0.8 and were resistant to three antibiotic classes only, whereas 18 cattle isolates had a MARI of 0.5-0.6 but no MDR detected (Table-4). In laying hens, 31/46 (67%) isolates had MDR for 3-7 antibiotic classes, and a MARI of 0.2-0.61. In swine, 20/24 (83%) isolates had MDR of 3-8 antibiotic classes and a MARI of 0.2-0.8.

**Table-3:** Multidrug resistance profile and resistance phenotype of *Enterococcus* spp. from cattle and sheep.

Isolate Code	Animal	Province	ATB	Class	Resistance phenotypic profile	Multiple antibiotic resistance index
M11 CIS	Sheep	NG	4	3	TEI+ERY+RIF+VAN	0.8
B32 MBE	Cattle	NG	3	2	TET+RIF+VAN	0.6
M1 CIS	Sheep	NG	3	2	TET+RIF+VAN	0.6
M3 CIS	Sheep	NG	3	3	ERY+RIF+VAN	0.6
B10 GAL	Cattle	NG	2	2	RIF+VAN	0.5
B11 GAL	Cattle	NY	2	2	RIF+VAN	0.5
B17 GAL	Cattle	NY	2	2	TEI+RIF	0.5
B4 SIA	Cattle	NY	2	2	RIF+ERY	0.5
B12 SIA	Cattle	NY	2	2	RIF+ERY	0.5
B13 SIA	Cattle	NY	2	2	RIF+VAN	0.5
B1 KOU	Cattle	NY	2	2	RIF+VAN	0.5
B5 MBE	Cattle	NG	2	2	RIF+VAN	0.5
B4 MBE	Cattle	NG	2	2	RIF+VAN	0.5
B28 MBE	Cattle	NG	2	2	RIF+VAN	0.5
B25 MBE	Cattle	NG	2	2	RIF+VAN	0.5
B20 MBE	Cattle	NG	2	2	RIF+VAN	0.5
B18 MBE	Cattle	NG	2	2	TEI+RIF	0.5
B17 MBE	Cattle	NG	2	2	RIF+VAN	0.5
B16 MBE	Cattle	NG	2	2	RIF+VAN	0.5
B14 MBE	Cattle	NG	2	2	RIF+ERY	0.5
B13 MBE	Cattle	NG	2	2	RIF+VAN	0.5
M5 IDJ	Sheep	NG	2	2	TET+RIF	0.5
M2 EUG	Sheep	NG	2	2	RIF+VAN	0.5
M7 MBE	Sheep	NG	2	2	RIF+VAN	0.5
M3 INC	Sheep	NG	2	2	RIF+VAN	0.5
M9 INC	Sheep	NG	2	2	RIF+ERY	0.5
M16 INC	Sheep	NG	2	2	RIF+ERY	0.5

TEI=Teicoplanin, ERY=Erythromycin, RIF=Rifampicin, VAN=Vancomycin, ATB=Antibiotics

**Table-4:** Multidrug resistance profile and resistance phenotype of *Enterococcus* from swine and laying hens.

Isolate code	Animal	Province	ATB	Class	Resistance phenotypic profile	Multiple antibiotic resistance index
N6GW P5	Swine	HO	8	7	TET+RIF+VAN+KNM+CEP+NOR+CIP+AMP	0.61
MAK P9	Swine	HO	8	7	TET+RIF+VAN+CHL+CEP+NOR+CIP+AMP	0.61
MAKP29	Swine	HO	7	6	TET+RIF+VAN+CEP+NOR+CIP+AMP	0.53
MAKP15	Swine	HO	7	5	TET+VAN+STR+KNM+CEP+CIP+NOR	0.53
FAENPP18	Laying yens	HO	7	5	TET+TEI+RIF+VAN+CEP+CIP+NOR	0.53
TITO PP22	Laying hens	HO	7	6	TET+RIF+ERY+STR+KNM+CEP+CIP	0.53
MAKP13	Swine	HO	7	7	TET+RIF+VAN+KNM+CEP+CIP+AMP	0.53
GRA PP30	Laying hens	HO	6	5	TET+VAN+ERY+STR+KNM+CEP	0.46
GRA PP25	Laying hens	HO	6	5	TET+VAN+STR+KNM+CEP+ERY	0.46
GRA PP25	Laying hens	HO	6	5	TET+VAN+ERY+STR+KNM+CEP	0.46
NGW P9	Swine	HO	6	5	TET+TEI+RIF+VAN+CEP+CIP	0.46
GRA PP6	Laying hens	ES	6	5	TET+ERY+STR+CEP+KNM+CIP	0.46
GRA PP16	Laying hens	ES	6	5	TET+ERY+STR+CEP+KNM+CIP	0.46
GRAPP18	Laying hens	ES	6	5	TET+ERY+STR+KNM+CEP+CIP	0.46
MAKP30	Swine	HO	6	5	RIF+VAN+STR+CEP+NOR+CIP	0.46
FAENPP16	Laying hens	HO	6	4	TET+TEI+VAN+NOR+CIP+AMP	0.46
MAKP16	Laying hens	HO	5	4	RIF+VAN+CEP+CIP+NOR	0.38
FAENPP11	Laying hens	HO	5	3	TEI+VAN+NOR+CIP+AMP	0.38
FAENPP8	Laying hens	HO	5	3	TEI+VAN+NOR+CIP+RIF	0.38
FAENPP25	Laying hens	HO	5	3	TEI+VAN+NOR+CIP+AMP	0.38
FAENPP3	Laying hens	HO	5	3	TEI+VAN+NOR+CIP+AMP	0.38
GRA PP15	Laying hens	HO	5	4	TET+VAN+ERY+STR+KNM	0.38
TITO PP13	Laying hens	HO	5	5	TET+RIF+ERY+CEP+CIP	0.38
TITO PP12	Laying hens	HO	5	4	TET+RIF+STR+KNM+CIP	0.38
FAEN PP1	Laying hens	HO	5	3	TEI+VAN+NOR+CIP+AMP	0.38
GRA PP4	Laying hens	ES	5	5	TET+ERY+STR+CEP+VAN	0.38
MAKP29	Swine	HO	5	5	TET+VAN+NOR+CHL+AMP	0.38
MAKP10	Swine	HO	5	5	TET+VAN+CEP+NOR+CIP	0.38
MAKP3	Laying hens	HO	4	3	VAN+CEP+CIP+NOR	0.3
GRA PP4	Laying hens	ES	4	4	TET+ERY+STR+CEP	0.3
GRA PP4	Laying hens	HO	4	4	TET+VAN+ERY+STR	0.3
TITO PP3	Laying hens	HO	4	3	TET+ERY+STR+KNM	0.3
GRA PP10	Laying hens	ES	4	4	TET+ERY+STR+CEP	0.3
NKIP5	Swine	ES	4	4	TET+VAN+STR+CEP	0.3
GRA PP26	Laying hens	ES	4	4	TET+VAN+ERY+CEP	0.3
GRA PP5	Laying hens	ES	4	4	TET+ERY+STR+CEP	0.3
NGWP11	Swine	HO	4	4	RIF+VAN+CEP+CIP	0.3
GRAPP11	Laying hens	ES	4	4	TET+ERY+STR+CEP	0.3
NGWP8	Swine	HO	4	4	TET+STR+CEP+CIP	0.3
TITO PP11	Laying hens	HO	3	2	TET+STR+KMN	0.2
TITO PP1	Laying hens	HO	3	2	STR+KNM+CEP	0.2
TITO PP8	Laying hens	HO	3	3	TET+RIF+STR	0.2
NKIP6	Swine	ES	3	3	TET+VAN+CEP	0.2
NKIP2	Swine	ES	3	3	VAN+STR+CEP	0.2
NKIP3	Swine	ES	3	3	VAN+STR+CEP	0.2
GRA PP2	Laying hens	ES	3	3	TET+STR+CEP	0.2
NKI P21/P3	Swine	ES	3	3	RIF+VAN+CIP	0.2
MAK P8	Swine	HO	3	3	VAN+CEP+CIP	0.2
NKI P18	Swine	ES	3	3	TET+VAN+CEP	0.2
MAK P2	Swine	HO	3	3	VAN+CEP+CIP	0.2
NGWP13	Swine	HO	3	3	VAN+CEP+CIP	0.2
GRAPP2	Laying hens	HO	3	3	TET+STR+CEP	0.2

TEI=Teicoplanin, ERY=Erythromycin, RIF=Rifampicin, VAN=Vancomycin, TE=Tetracycline, STR=Streptomycin, KMN=Kanamycin, CEP=Cephalothin/cephalexin, CHL=Chloramphenicol, AMP=Ampicillin, NOR=Norfloxacin, CIP=Ciprofloxacin, ATB=Antibiotics

MARI ranged 0.61-0.2 in the Haut-Ogooué and 0.46-0.2 in Estuaire (Table-4) whereas from 0.8-0.5 in Ngounié and was 0.6 in Nyanga (Table-3). Twenty-four isolates were MDR in Haut-Ogooué, while 16 isolates were MDR in Estuaire. Two MDR isolates were obtained in Ngounie and none in Nyanga (Table-3).

## Discussion

### Distribution of enterococci

Antibiotic resistance is a major problem in the world today in the environment, animals, and humans. The emergence of resistance in livestock is most often the result of antibiotic consumption [20]. Studies of resistance in livestock are important to assess the emergence and spread of resistance in farms. This study investigated data on antibiotic resistance in the *Enterococcu*s genus in Gabonese livestock. The high prevalence of these bacteria was obtained in all species studied. Our results are not surprising as several studies have shown a similar prevalence in farm animals [24-26] and *Enterococcus* is ubiquitous in humans, animals, and in the environment [13,27].

### Resistance prevalence Enterococci

A high prevalence of resistance to CEP (68%) and moderate for STR (49%) was obtained in enterococcal isolates. These results are similar to those from South Africa [20,28], Nigeria [29], and Angola [30] and could be explained by intrinsic resistance to clinically achievable concentrations of these antibiotics in enterococci [31]. In fact, Enterococci exhibit intrinsic resistance for cephalosporins caused by low expression of penicillin-binding proteins and poor uptake of antibiotics, enzyme-mediated resistance, or sterically hindered ribosome target sites for aminoglycoside.

Among the other resistances observed, a high prevalence of resistance to RIF (57%) and moderate to VAN (35%) and ERY (16%) was found. Similar results were obtained in Tanzanian [32,33] and Nigerian [34] livestock for RIF. Several authors have suggested that this resistance could be the result of the transmission of bacteria from humans to farm animals through the consumption of human waste or contaminated water when the animals roam or during transhumance [32,35,36]. The *rpo*B gene encoding the b subunit of RNA polymerase is responsible for observed resistance to RIF [37]. For resistance to VAN and ERY, it should be remembered that these antibiotics are not used in veterinary medicine in Gabon. However, they have been linked to the emergence of resistance in Europe due to their use as a growth promotor in European livestock [38-41]. The mechanism of resistance to glycopeptides relies on binding to the D-Ala-D-Ala pentapeptide terminus which binds to VAN, thereby modifying the terminus to D-Ala-D-Lac or to D-Ala-D-Ser [42]. Resistance to MLS_B_ is through three mechanisms: methylation of 23SrRNA, active efflux, and inactivating enzymes [31]. The *erm*B and *van*C gene is the most common acquired resistance to ERY and VAN in African livestock [5,43]. This result could be due to persistence of resistance in animals originating from Europe, where a high prevalence of this resistance was observed in the previous years [40-41].

TET, which is widely used in veterinary medicine due to its broad spectrum of action on a variety of pathogens, had a moderate prevalence of resistance in our study (20%). This prevalence is high compared to studies in Nigeria [44] and Ethiopia [45] and may be related to the frequent use of these drugs in veterinary medicine, which could increase the number of resistant strains [46]. The main mechanisms of resistance include efflux pumps, modification of ribosomal RNA, and inactivation of the antibiotic. The *tet*(M) gene coding for ribosomal protection is frequently detected in African livestock [47-49].

### Prevalence of resistance in enterococci by province

The identification of areas where resistance is emerging is an important step in the investigation of resistance for antibiotics used in veterinary medicine [50]. In our study, a diversity of antibiotic resistance was observed in the provinces of Haut-Ogoouée and Estuaire. These results could be related to the higher number of livestock found in these provinces due to the high demand for animal protein [51,52]. The use of antibiotics in animal husbandry is more important in places with high human density and directly correlates with resistance in livestock.

### Antibiotic susceptibility of the genus Enterococcus in farm animals

In Gabon, the most exploited livestock are chickens, swine, sheep, and cattle. In this study, CIP resistance was moderate for laying hens (32%) and high for swine (68%). Similar prevalences have been observed in Tunisia [49] and South Africa [28]. These results are surprising because quinolones are not indicated for the treatment of infections in Gabonese livestock. This result could be due to the transfer of mobile genetic elements in the intestinal tract of animals leading to acquired resistance. AMP showed moderate resistance in laying hens (15%) and swine (14%). Similar results were obtained in Tanzanian livestock [35]. AMP is used to treat Enterococci infection (e.g., urinary tract infection or non-endocarditis infection) in hospitals [53]. A high rate of resistance to VAN was linked in all species of animals studied. It is necessary to determine its resistance by other tests (minimum inhibitory concentration and PCR test) to confirm resistance to the glycopeptides. However, *E. gallinarum* and *E. casseliflavus* species carry acquired resistance to VAN. A species description would be necessary to confirm the absence of these two species in our study.

### Phenotypic profile of resistance

MARI and MDR were higher in laying hens and swine in our study and in Egyptian [54] and Nigerian livestock [25]. This result could be due to the health requirement of these animals compared to cattle and small ruminants, which are grazing animals compared to swine and laying hens. Haut-Ogooué and Estuaire had higher indexes and multiple resistances than the provinces of Nyanga and Ngounié. This result reflects the high use of antibiotics in these provinces with a high risk of contamination in humans or the environment such as wastewater [54,55]. Laying hens and swine in Gabon present in the Estuaire and Haut-Ogouée provinces could be considered as reservoirs of antibiotic resistance genes.

## Conclusion

This study investigated antibiotic resistance in livestock farms in Gabon. In sum, Gabonese livestock can be considered potential reservoirs of resistance genes that could be disseminated in the environment. Our study complements data characterizing resistance in humans, animals, and the Gabon environment. Molecular characterization of the resistance obtained would allow a better description of the circulation of resistance genes. Description of the species of *Enterococcus* spp. associated with the various resistances would also be useful.

## Authors’ Contributions

OED: Conceptualization of the study, investigation, data curation, formal analysis, and methodology. BL and MMR: Review and editing of the manuscript. OR: Supervision of the study. KBS: Project administration. All authors read and approved the final manuscript.
